# Favorable Biochemical Freedom From Recurrence With Stereotactic Body Radiation Therapy for Intermediate and High-Risk Prostate Cancer: A Single Institutional Experience With Long-Term Follow-Up

**DOI:** 10.3389/fonc.2020.01505

**Published:** 2020-09-25

**Authors:** Anthony Ricco, Gabrielle Barbera, Rachelle Lanciano, Jing Feng, Alexandra Hanlon, Alicia Lozano, Michael Good, Stephen Arrigo, John Lamond, Jun Yang

**Affiliations:** ^1^Virginia Commonwealth University Health System, Richmond, VA, United States; ^2^College of Medicine, Drexel University, Philadelphia, PA, United States; ^3^Radiation Oncology, Crozer-Keystone Health System, Springfield, PA, United States; ^4^Philadelphia CyberKnife Center, Havertown, PA, United States; ^5^Virginia Polytechnic Institute and State University, Blacksburg, VA, United States

**Keywords:** prostate cancer, SBRT (stereotactic body radiation therapy), high risk prostate cancer, prostate SBRT treatment, intermediate risk prostate cancer

## Abstract

**Purpose/Objective(s):** The current study reports long-term overall survival (OS) and biochemical freedom from recurrence (BFFR) after stereotactic body radiation therapy (SBRT) for men with intermediate and high-risk prostate cancer in a single community hospital setting with early adoption.

**Materials/Methods:** Ninety-seven consecutive men with intermediate and high-risk prostate cancer treated with SBRT between 2007 and 2015 were retrospectively studied. Categorical variables for analysis included National Comprehensive Cancer Network risk group, race, Gleason grade group, T stage, use of androgen deprivation therapy, and planning target volume dose. Continuous variables for analysis included pretreatment prostate-specific antigen (PSA), percent cores positive, age at diagnosis, PSA nadir, prostate volume, percent prostate that received 40 Gy, and minimum dose to 0.03 cc of prostate (Dmin). BFFR was assessed using the Phoenix nadir +2 definition. OS and BFFR were estimated using Kaplan–Meier (KM) methodology with comparisons accomplished using log-rank statistics. Multivariable analysis (MVA) was accomplished with a backwards selection Cox proportional-hazards model with statistical significance taken at the *p* < 0.05 level.

**Results:** Median FU is 78.4 months. Five- and ten-year OS KM estimates are 90.9 and 73.2%, respectively, with 19 deaths recorded. MVA reveals pretreatment PSA (*p* = 0.032), percent prostate 40 Gy (*p* = 0.003), and race (*p* = 0.031) were predictive of OS. Five- and nine-year BFFR KM estimates are 92.1 and 87.5%, respectively, with 10 biochemical failures recorded. MVA revealed PSA nadir (*p* < 0.001) was the only factor predictive of BFFR. Specifically, for every one-unit increase in PSA nadir, there was a 4.2-fold increased odds of biochemical failure (HR = 4.248). No significant differences in BFFR were found between favorable intermediate, unfavorable intermediate, and high-risk prostate cancer (*p* = 0.054) with 7-year KM estimates of 96.6, 81.0, and 85.7%, respectively.

**Conclusions:** Favorable OS and BFFR can be expected after SBRT for intermediate and high-risk prostate cancer with non-significant differences seen for BFFR between favorable intermediate, unfavorable intermediate, and high-risk groups. Our 5-year BFFR compares favorably with the HYPO-RT-PC trial of 84%. PSA nadir was predictive of biochemical failure. This study is ultimately limited by the small absolute number of high-risk patients included.

## Introduction

Owing to advancements in imaging and treatment technologies over the past three decades leading to lower rectal and bladder doses, along with radiobiologic, cost, and convenience rationale, hypofractionated radiotherapy schedules for prostate cancer have increasingly been studied. Moderate hypofractionation is now the standard of care per National Comprehensive Cancer Network (NCCN) guidelines and a joint American Society for Radiation Oncology, American Urological Association, and American Society of Clinical Oncology guideline (1).

In a rapidly changing research landscape, SBRT has demonstrated similar toxicity profiles and non-inferior disease control compared with conventionally fractionated radiotherapy in many phase I/II trials (2–17), a landmark phase III trial (18), a National Cancer Database propensity matched analysis (19), and a recently completed systematic review and meta-analysis (20). Included in these trials were intermediate and high-risk patients; however, not all studies provided biochemical or overall survival data as stratified by risk group and high-risk patients made up only a minority of patients. Despite the rapid change in published literature supporting SBRT in all risk groups, consensus guidelines have lagged with SBRT recommended on a clinical trial or registry for unfavorable intermediate and high-risk prostate cancer (1).

In the current study, we provide long-term biochemical freedom from recurrence and overall survival outcomes for NCCN favorable intermediate, unfavorable intermediate, and high risk localized prostate cancer treated with SBRT in a single community hospital setting with early adoption of this technology.

## Methods

Our objective was to examine the relationships between demographic and clinical factors with two primary outcomes among intermediate and high-risk prostate cancer patients: overall survival (OS) and biochemical freedom from recurrence (BFFR). This review represents an update of our previously reported results.

Ninety-seven consecutive men treated with SBRT between 2007 and 2015 were retrospectively studied on this IRB-supported trial (CK18-001). Categorical variables for analysis included NCCN risk group, race, Gleason grade group, T stage, use of androgen deprivation therapy (ADT), and planning target volume (PTV) dose. Continuous variables for analysis included pretreatment prostate-specific antigen (PSA), percent cores positive, age at diagnosis, PSA nadir, prostate volume, percent prostate that received 40 Gy, and minimum dose to 0.03 cc of prostate (Dmin). The majority of patients had sextant or extended biopsies; however, saturation biopsies were rare.

All patients had MRI and CT treatment planning images with 1-mm slice thickness merged on fiducials for prostate and seminal vesicle contouring. Two target doses were prescribed: 40 Gy to the prostate and 36.25 Gy to the prostate +1–2 cm of seminal vesicle with 3 mm margin posteriorly and 5 mm elsewhere. Hollow viscera were contoured as solid structures. Dose–volume histogram analysis constrained normal tissue doses as follows: volume of rectum receiving more than 36 Gy (rectum V36) <1 cm^3^; bowel V30Gy <1 cm^3^; penile bulb V29.5Gy <50%; bladder V37Gy <5–10 cm^3^ (over time decreased to V37 <2 cm^3^). The urethra was not typically contoured with no dose constraint used. SBRT was delivered with the CyberKnife system in 5 fractions usually with fractions delivered every other day using Iris variable collimators and typically 200–250 non-coplanar beams. Orthogonal X-ray image pairs were obtained throughout treatment for use in motion management. The real-time prostate position was locked on by the relative fiducial position on the X-rays. For those patients with evenly distributed fiducials in the prostate quadrants, the prostate's rotation was also tracked and corrections were made in real time. We participated in a multicenter national trial of SBRT for low and intermediate-risk prostate cancer and followed the aforementioned protocol guidelines for all patients (17). Treatment plans were reviewed in Multiplan for this study to obtain additional dosimetric data that could be prognostic such as percent prostate that received 40 Gy and minimum dose to 0.03 cc of prostate (Dmin). Five of the oldest patient plans were not available for dosimetric review.

BFFR was assessed using the Phoenix nadir +2 definition. BFFR was analyzed at the patient level and was defined as years from end of SBRT to biochemical failure or most recent PSA for patients who did not have a biochemical recurrence. The analytic sample for this outcome included 97 patients for all strata, except for percent of gross tumor volume (GTV) receiving 40 Gy and GTV Dmin (*n* = 92) owing to missing data. Of note, there were 10 failures among 97 patients in this study, giving us the power to detect at most one predictor in the final multivariable model based on rule of thumb.

OS was analyzed at the patient level and was defined as years from end of SBRT to death or most recent follow-up (FU) for patients who survived. The analytic sample for the overall survival outcome included 97 patients for all strata, except for percent of GTV receiving 40 Gy and GTV Dmin (*n* = 92) owing to missing data. Of note, there were 19 deaths among the 97 patients in this study, giving us the power to detect two to three predictors in the final multivariable model based on rule of thumb.

Patient characteristics, including demographic (age, race) and clinical factors (risk group, Gleason grade group (GGG), T stage, pre-treatment PSA, percent cores positive, use of hormone therapy, PSA nadir value, PTV dose, prostate volume, percent of prostate receiving 40 Gy, GTV Dmin) were described using medians, SDs, frequencies, and percentages. OS and BFFR were estimated using Kaplan–Meier (KM) methodology with comparisons accomplished using log-rank statistics. Bivariate Cox proportional-hazards (PH) models were used to examine the individual impact of each demographic and clinical factor on OS and BFFR. Variables demonstrating significance at the *p* < 0.20 level in the bivariate models were included into a backwards selection multivariable Cox PH model for each outcome. Predictors were subsequently removed one at a time based on the largest *p* values until all variables remaining in the final model for each outcome were significant at the *p* < 0.05 level. All statistical analyses were performed using SAS V.94 (SAS Institute, Cary, NC, USA). Statistical significance was taken at the *p* < 0.05 level.

## Results

### Patient Characteristics

Patient characteristics of 97 patients with complete data for OS and BFFR are summarized in Tables 1, 2. Patients were predominantly white (60.8%), with a median age of 68.0 years (Q1:Q3 63–73). The majority of patients were stage T1c (65.0%), had no hormonal therapy (60.8%), and received a dose of 36.25 Gy (82.5%). Most patients were considered unfavorable intermediate risk (45.4%, *n* = 44) and had GGG equal to 2 (47.4%, *n* = 46). In addition, in this sample, patients had median pretreatment PSA of 6.2 (Q1:Q3 4.82–10.60), median percent cores positive of 33.33% (Q1:Q3 16.67–50%), median prostate volume of 52.76 cc (Q1:Q3 38.91–75.25), median percent of prostate receiving GTV 40 Gy of 85% (Q1:Q3 80.35–90.15%), median Dmin GTV of 36.74 Gy (Q1:Q3 36.08–37.18Gy), and median PSA nadir of 0.1 (Q1:Q3 0.1–0.27). Median follow-up was 78.4 months.

**Table 1:** Descriptive statistics for continuous variables.

**Variable**	**N**	**Mean**	**SD**	**Median**	**Interquartile range**		**Range**	
					**Q1**	**Q3**	**Min**	**Max**
Pre-treatment PSA	97	9.36	8.87	6.20	4.82	10.60	1.27	62.00
% Cores positive	97	33.93	21.05	33.33	16.67	50.00	6.26	100
Age at diagnosis	97	68.03	7.14	68.00	63.00	73.00	53.00	86.00
PSA nadir value	97	0.31	0.59	0.10	0.10	0.27	0.00	4.10
Prostate volume (cc)	97	60.85	31.05	52.76	38.91	75.25	13.83	162.23
% of Prostate 40 Gy	92	81.30	18.95	85.00	80.35	90.15	0.00	98.30
GTV min (0.03 cc) (Gy)	92	35.93	4.02	36.74	36.08	37.18	7.16	38.41
Overall survival (years)	97	6.57	2.68	6.53	4.53	8.56	1.52	11.48
Biochemical freedom from recurrence (years)	97	5.63	2.79	5.96	3.23	7.68	0.71	11.15

*GTV, gross tumor volume; PSA, prostate-specific antigen*.

**Table 2:** Descriptive statistics for categorical variables.

**Variable**	***n***	**(%)**
**Race**		
Asian	2	2.06
Black	34	35.05
Egyptian	1	1.03
Hispanic	1	1.03
White	59	60.82
**Risk group**		
Intermediate favorable	42	43.30
Intermediate unfavorable	44	45.36
High	11	11.34
**GGG**		
1	17	17.53
2	46	47.42
3	29	29.90
4	5	5.15
**T stage**		
T1c	63	64.95
T2a	15	15.46
T2b	9	9.28
T2c	10	10.31
**Use of hormone therapy**		
Yes	38	39.18
No	59	60.82
**Dose**		
35	2	2.06
36.25	80	82.47
37.5	15	15.46

*GGG, Gleason grade group*.

### Overall Survival

**Figure 1:**
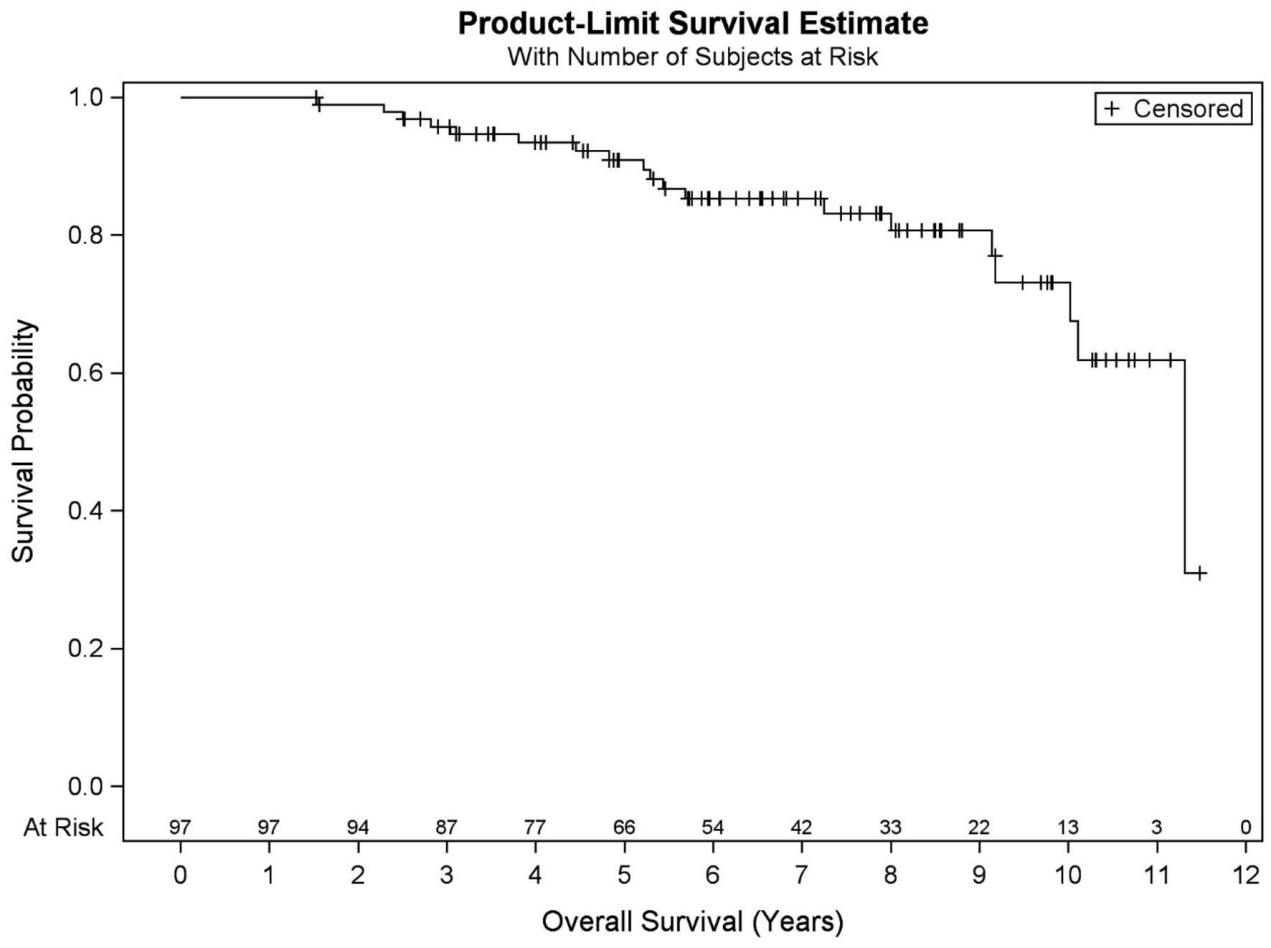
Kaplan–Meier curve for overall survival in all patients (*N* = 97).

**Figure 2:**
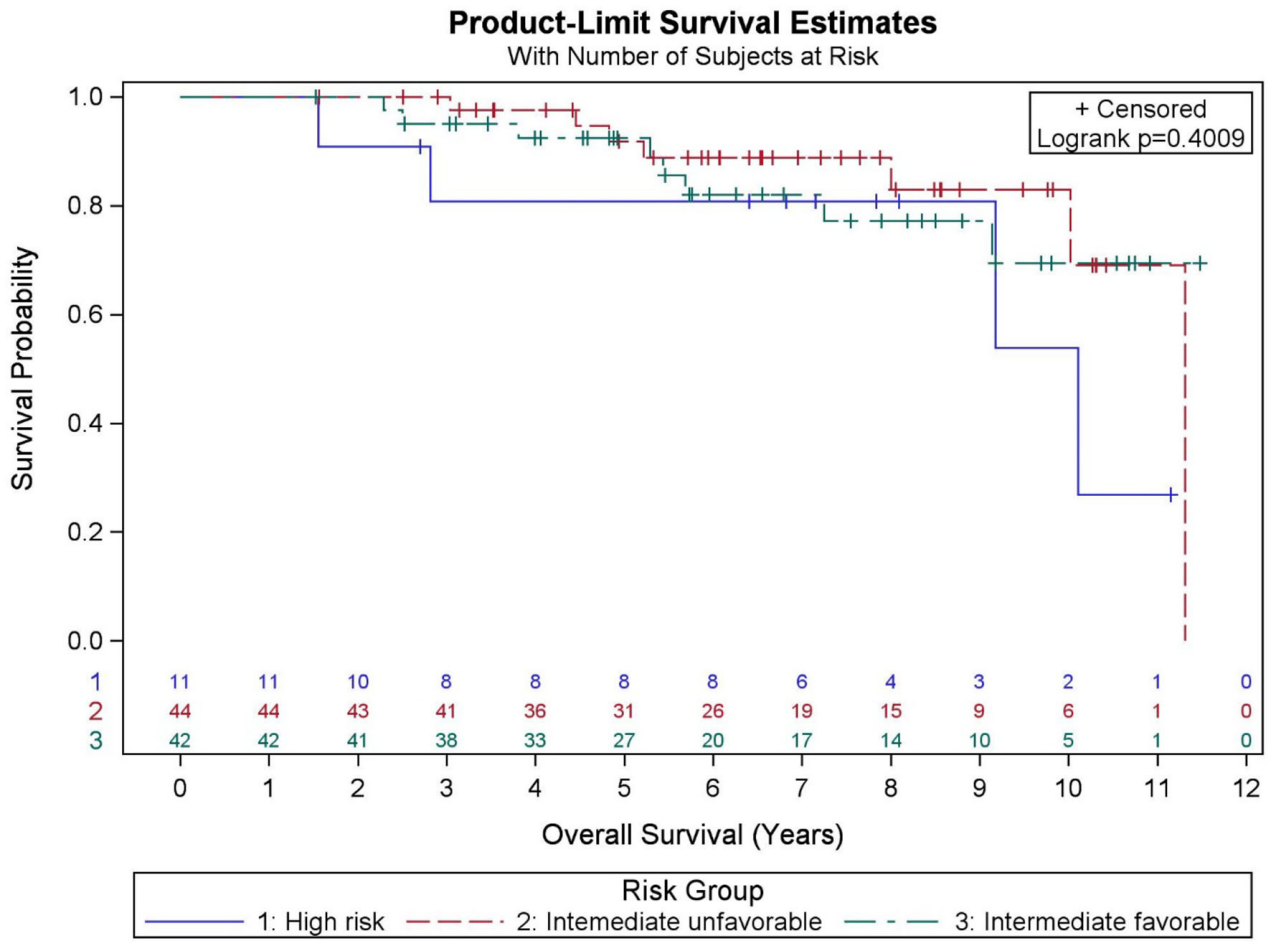
Kaplan–Meier curves for overall survival by risk group.

Among all patients (*N* = 97), KM OS estimates at 5 and 10 years were 90.9 and 73.2%, respectively (Figure 1). No significant differences in OS were found between favorable intermediate, unfavorable intermediate, and high-risk prostate cancer (*p* = 0.401). The 7-year KM estimates for OS for favorable intermediate, unfavorable intermediate, and high-risk prostate cancer were 82.0, 88.9, and 80.8%, respectively (Figure 2).

Age at diagnosis (*p* = 0.032), race (*p* = 0.029), pre-treatment PSA (0.046), dose (*p* = 0.084), prostate volume (*p* = 0.170), percent of prostate receiving 40 Gy (*p* = 0.006), and Dmin (*p* = 0.077) were significantly associated with OS at the *p* < 0.20 level in the bivariate models and were considered in the final multivariable model. Results from a backwards selection multivariable Cox PH model demonstrated that only pretreatment PSA (*p* = 0.032), percent of prostate receiving 40 Gy (*p* = 0.003), and race (*p* = 0.031) were found to be predictive of OS. Specifically, for every one-unit increase in pre-treatment PSA, the odds of death increased by 4% (HR = 1.040, 95% CI = 1.003–1.077). In addition, the odds of death increased by 2.3% for each one-unit decrease in percent of prostate receiving 40 Gy (HR = 0.977, 95% CI = 0.961–0.992). Lastly, white patients had 4.1-fold increased odds of death as compared with non-white patients (HR = 4.068, 95% CI = 1.140–14.512).

### Biochemical Freedom From Recurrence

**Figure 3:**
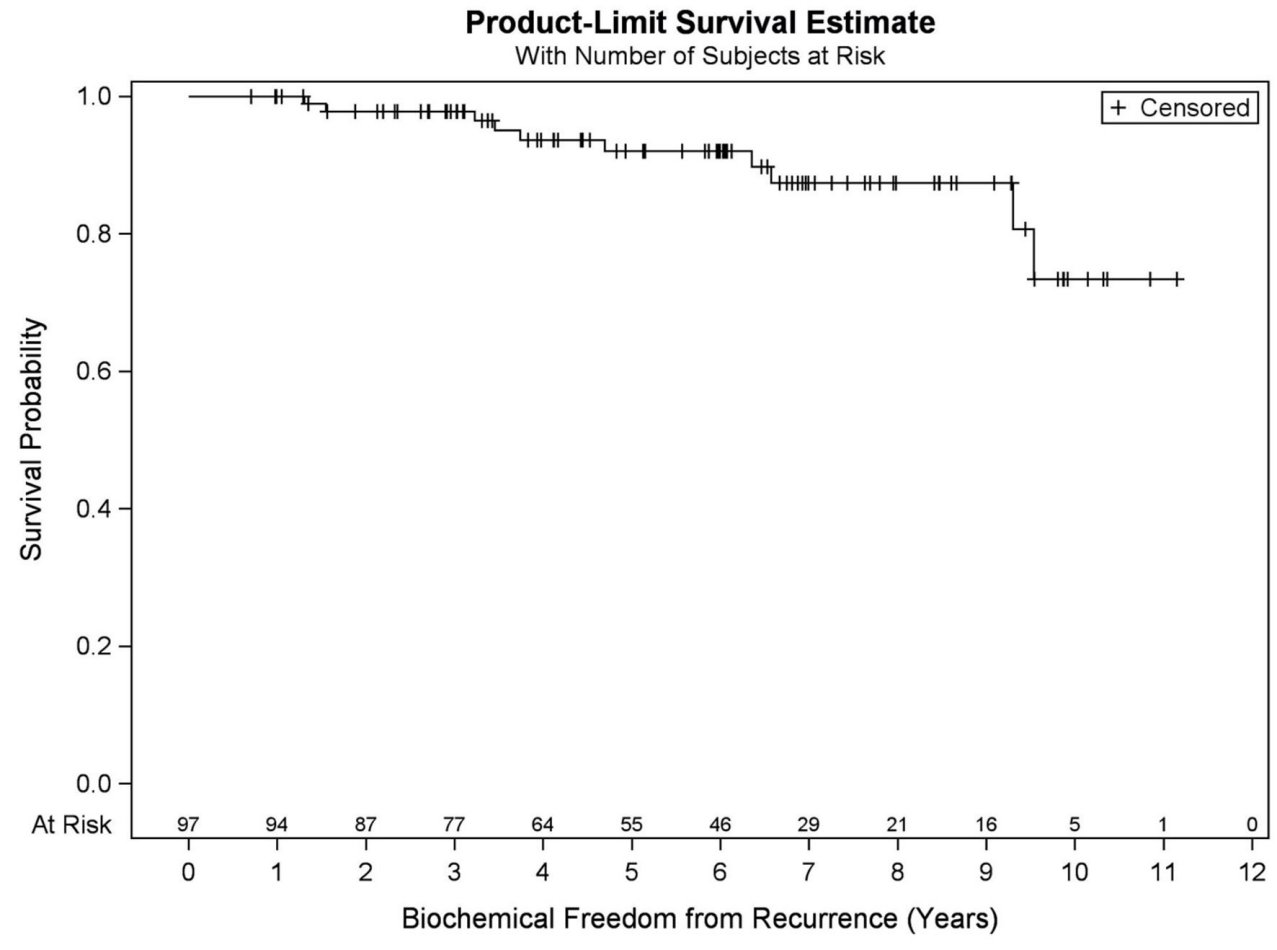
Kaplan–Meier curves for biochemical freedom from recurrence in all patients (*N* = 97).

**Figure 4:**
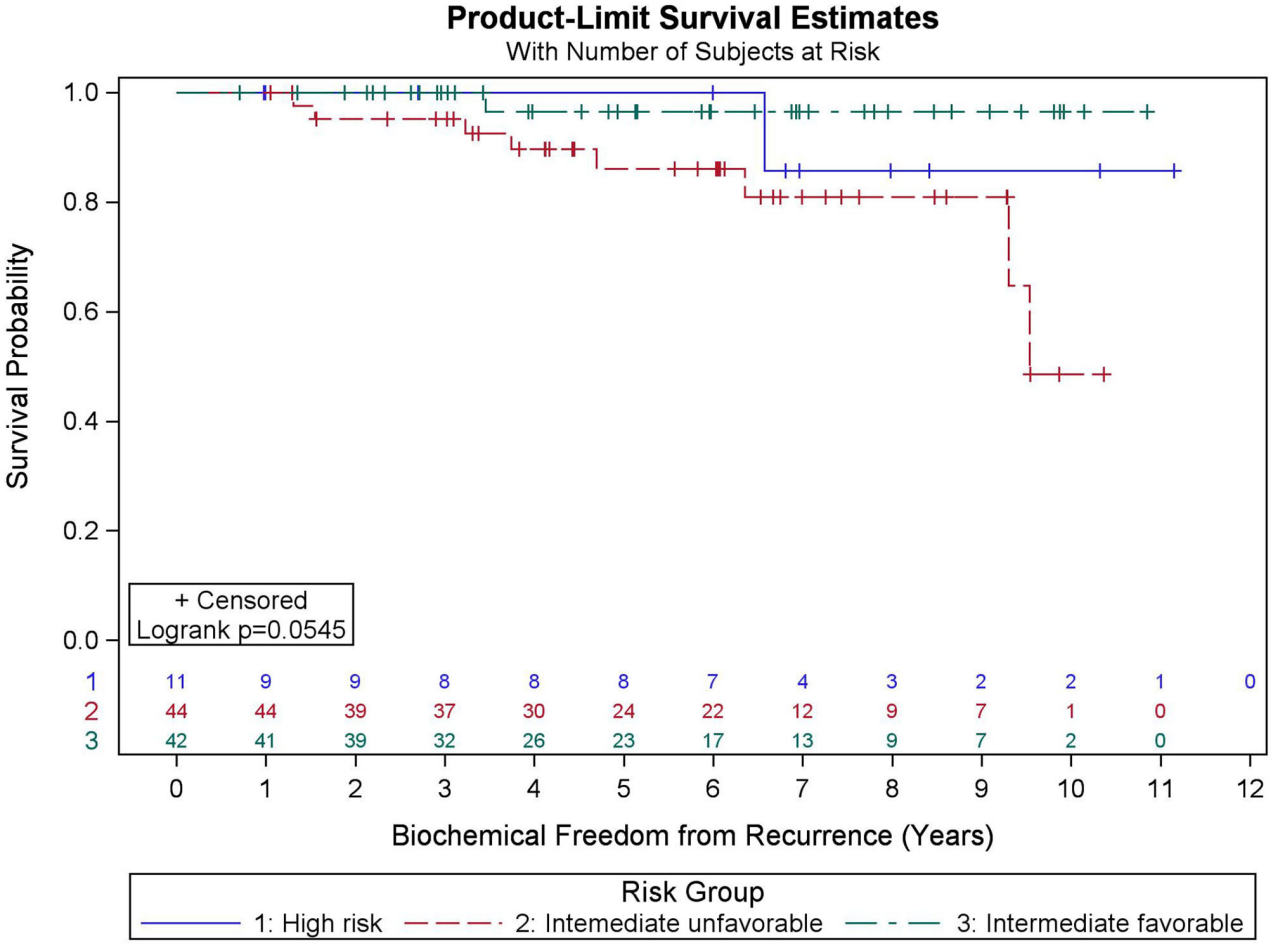
Kaplan–Meier curves for biochemical freedom from recurrence by risk group.

Among all patients (*N* = 97), KM BFFR estimates at 5 and 9 years were 92.1 and 87.5%, respectively (Figure 3). No significant differences in BFFR were found between favorable intermediate, unfavorable intermediate, and high-risk prostate cancer (*p* = 0.054-log rank). The 7-year KM estimates for BFFR for favorable intermediate, unfavorable intermediate, and high-risk prostate cancer were 96.6, 81.0, and 85.7%, respectively (Figure 4).

**Figure 5:**
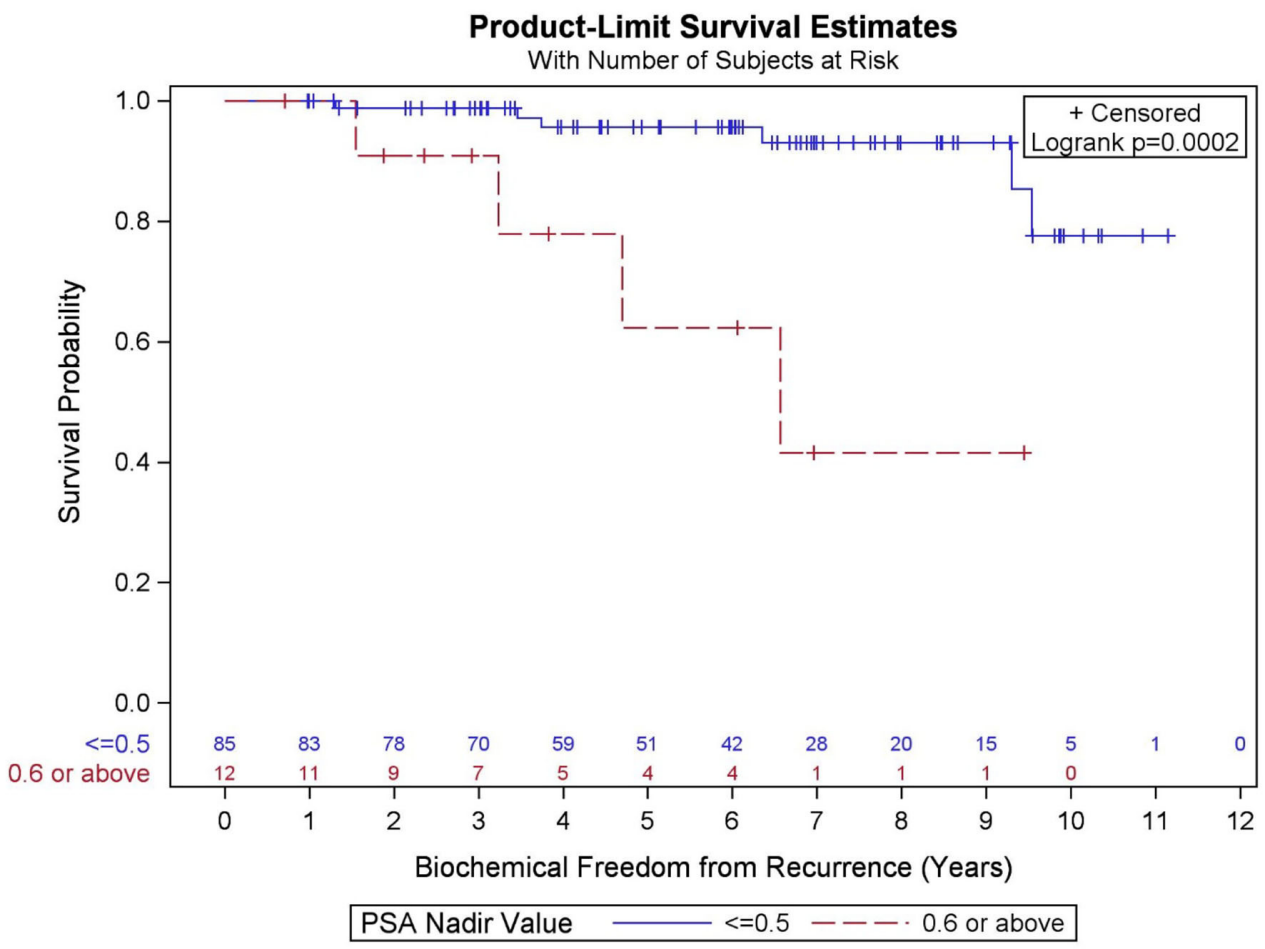
Kaplan–Meier curves for biochemical freedom from recurrence by prostate-specific antigen (PSA) nadir value.

GGG (*p* = 0.095), PSA nadir value (Figure 5, *p* < 0.001), and percent of prostate receiving 40 Gy (*p* = 0.133) were significantly associated with BFFR at the *p* < 0.20 level in the bivariate models and were considered in the final multivariable model. Results from a backwards selection multivariable Cox PH model demonstrated that only PSA nadir (*p* < 0.001) was found to be predictive of BFFR. Specifically, for every one-unit increase in PSA nadir, there was 4.2-fold increased odds of biochemical failure (HR = 4.248, 95% CI = 2.236–8.073).

No statistically significant differences in BFFR were observed for risk group, race, T stage, pre-treatment PSA, percent cores positive, age, use of hormone therapy, PTV dose, prostate volume, and GTV Dmin. In addition, there was no significant association between percent of prostate receiving 40 Gy and PSA nadir (*p* = 0.26).

### Patterns of Failure

In total, there were 19 deaths during the follow-up period, of which only one patient died of prostate cancer. There were three patients who developed metastatic disease: one with lung metastasis alive with disease, one with bony metastases and pelvic lymph node metastasis alive with disease, and one with unknown site of metastatic disease who died from disease. There was only one patient with documented local only failure with seminal vesicle recurrence.

## Discussion

Herein we present our long-term biochemical freedom from recurrence and overall survival outcomes for NCCN favorable intermediate, unfavorable intermediate, and high-risk localized prostate cancer treated with SBRT, with favorable results in this single-institution retrospective cohort. Our biochemical freedom from recurrence outcomes at 7 years across risk groups compare favorably with other published series, as discussed later. Our median survival and follow-up represents one of the longest reported in the literature (21).

As increasing evidence supports the use of SBRT for localized prostate cancer with similar rates of biochemical freedom from recurrence, acute/late toxicity, and patient quality of life outcomes compared with conventional fractionation or moderate hypofractionation, SBRT has been an available standard-of-care treatment option for low and favorable intermediate-risk patients. NCCN guidelines have recently included SBRT as acceptable treatment for unfavorable intermediate or high-risk prostate cancer (22). This acceptance is however conditional and “SBRT can be considered if delivering longer courses of external beam radiation would present medical or social hardship.”

A recently completed meta-analysis of 38 prospective series of SBRT included over 2,900 patients with intermediate-risk disease reporting 5-year BFFR of 92.1% (20). These outcomes were not stratified by favorable or unfavorable intermediate risk groups, however. Available evidence suggests equivalent outcomes for SBRT for men with unfavorable intermediate-risk prostate cancer compared with conventionally fractionated radiation. The recently published HYPO-RT-PC randomized phase III non-inferiority trial included 1,200 patients, of which 89% were intermediate risk and showed identical 5-year failure-free survival between ultra-hypofractionated and conventionally fractionated radiation (18). A recent article of completed prospective trials that pooled results for low and intermediate-risk patients did stratify results into favorable and unfavorable intermediate-risk groups. The 7-year BFFR rate was 93 and 85% for favorable intermediate and unfavorable intermediate-risk groups, respectively (10). This excellent BFFR rate is confirmed by another multicenter prospective trial, with 5-year rates of 100 and 93.1%, respectively, for favorable intermediate and unfavorable intermediate-risk patients (17). These control rates are comparable with our data, which are 96.6 and 81% at 7 years, respectively. In addition, the previously discussed results from pooled trials also support low rates of distant metastatic disease (1.7 and 3.0%, respectively), with no patients dying of prostate cancer (10). In our own analysis, three patients developed metastatic disease with one of those patients having prostate cancer–specific mortality.

Data supporting the use of SBRT in high-risk prostate cancer are more limited. The meta-analysis discussed previously also included 470 patients with high-risk disease, but was unable to provide a high-risk cohort estimate of biochemical freedom from recurrence given that many of the included studies did not provide estimates based on risk group (20). Our study included a minority with high risk (11.3%, *n* = 11), very similar to the proportion of patients in this meta-analysis (20). Numerous retrospective/single institution experiences have included high-risk patients from 7.4 to 65.9% of their patient cohorts, with variable total doses (32–40 Gy in 5 fractions) and ADT use (4, 23–34). In addition, a large National Cancer Database study did not find a difference in overall survival between SBRT and conventionally fractionated patients when propensity-matched high-risk subpopulations of Gleason score 8+ or PSA >10 (19). Despite potential selection bias, results are promising with 5-year BFFR between 70 and 80% in most series.

One of the largest series of high-risk patients (*n* = 52) with long follow-up (median 60 months) was the Katz et al. study which reported 6-year BFFR of 69%, comparable with the 7-year rate of 68% in high-risk patients treated on a dose-escalated conventionally fractionated trial (35, 36). The HYPO-RT-PC trial also included 11% (126 patients) with high-risk disease but did not stratify their biochemical outcomes by risk group. Given the small proportion of high-risk patients, the authors concluded that there is not enough evidence to support SBRT as standard of care for high-risk patients contrary to their conclusion for intermediate-risk patients. In addition, this trial did not use androgen deprivation therapy, which is standard of care for high-risk patients (18).

In the current study, PSA nadir >0.6 was highly predictive of biochemical failure. This is consistent with the previous literature, which supports that PSA nadir at a threshold below 0.5 predicts long-term clinical outcomes including biochemical freedom from recurrence, development of distant metastases, and OS (37–42). It is known that PSA decay after SBRT is similar to conventional fractionation through 2.75 years, at which point conventional radiation's decay rate plateaus and SBRT's rate continues a slow decline (43). It is unknown whether this represents depletion of further malignant cells or rather benign epithelial cells, and whether there might be long-term benefit to SBRT over conventionally fractionated radiotherapy given this favorable prognostic factor (43). In a separate analysis, we could not confirm an association between percent of prostate receiving 40 Gy and PSA nadir.

This study is ultimately limited by its retrospective nature and small absolute number of high-risk patients included. The power of this study is long-term follow-up in a single institution where SBRT was adopted early. There remain unresolved questions regarding SBRT in intermediate and high-risk patients, including optimal dose, dose constraints, use of ADT, use of nodal radiation, and patient selection criteria such as large prostate size, previous transurethral resection of the prostate, and large paramedian lobes (44–48). We hope our results can contribute to the growing body of data for intermediate and high-risk patients using SBRT as a standard treatment modality for localized prostate cancer.

## Data Availability Statement

The raw data supporting the conclusions of this article will be made available by the authors, without undue reservation.

## Ethics Statement

The studies involving human participants were reviewed and approved by Crozer Keystone Health System Institutional Review Board. Written informed consent for participation was not required for this study in accordance with the national legislation and the institutional requirements.

## Author Contributions

AR, GB, and RL collected data and organized into a written manuscript. SA, JL, JY, and JF all provided editorial support. AL and AH provided statistical support. All authors contributed to the article and approved the submitted version.

## Conflict of Interest

The authors declare that the research was conducted in the absence of any commercial or financial relationships that could be construed as a potential conflict of interest.
